# Neuromedin U promotes human type 2 immune responses

**DOI:** 10.1038/s41385-022-00543-6

**Published:** 2022-07-09

**Authors:** Yuan Ye, Jian Luo, Ni Zeng, Shan Jiang, Wentao Chen, Ryan D. Hoyle, Paul Klenerman, Ian D. Pavord, Luzheng Xue

**Affiliations:** 1 Respiratory Medicine Unit and NIHR Oxford Biomedical Research Centre, University of Oxford, Oxford, UK; 2 Translational Gastroenterology Unit and Peter Medawar Building for Pathogen Research, University of Oxford, Oxford, UK

## Abstract

Type 2 immunity mediates the immune responses against parasites and allergic stimuli. Evidence from studies of cell lines and animals implies that neuromedin U (NmU) acts as a pro-inflammatory mediator of type 2 inflammation. However, the role of NmU in human type 2 immunity remains unclear. Here we investigated the expression of NmU in human blood and airways, and the expression of NmU receptors by human immune cells in blood and lung tissue. We detected human NmU (hNmU-25) in blood and airways with higher concentrations in the latter. NmU receptor 1 (NmUR1) was expressed by most human immune cells with higher levels in type 2 cells including type 2 T helpers, type 2 cytotoxic T cells, group-2 innate lymphoid cells and eosinophils, and was upregulated in lung-resident and activated type 2 cells. We also assessed the effects of NmU in these cells. hNmU-25 elicited type 2 cytokine production by type 2 lymphocytes and induced cell migration, including eosinophils. hNmU-25 also enhanced the type 2 immune response to other stimuli, particularly prostaglandin D_2_. These results indicate that NmU could contribute to the pathogenic processes of type 2 immunity-mediated diseases in humans via its pro-inflammatory effects on type 2 lymphocytes and eosinophils.

## Introduction

Type 2 immunity not only confers host defenses against helminth infection but also mediates allergic inflammation induced by multiple stimuli^1^. The process has a key role in the pathogenesis of exacerbations of asthma and COPD^2,3^, chronic rhinosinusitis and nasal polyposis, atopic dermatitis, and eosinophilic gastrointestinal disorders^4^. The hallmark of adaptive type 2 responses is the induction of type 2 cytokines including interleukin (IL)-4, IL-5 and IL-13, which are mainly released by type 2 T helpers (Th2), type 2 cytotoxic T cells (Tc2) and group 2 innate lymphoid cells (ILC2)^5–7^. Besides the regulation of type 2 immunity by various inflammatory mediators such as lipid mediators prostaglandin D_2_ (PGD_2_), cysteinyl leukotriene E_4_ (LTE_4_) and epithelial alarmins IL-25, IL-33 and thymic stromal lymphopoietin (TSLP), increasing evidence indicates that neuropeptides also contribute to type 2 inflammation potentially reflecting neuro-immune interactions^8^. For example, neuropeptide calcitonin gene-related peptide enhances type 2 immunity by promoting the functional effects of Langerhans cell on Th2 responses^9^. Calcitonin gene-related peptide and vasoactive intestine peptide were also reported to facilitate ILC2-induced type 2 inflammation^10,11^.

Recently, neuromedin U (NmU), a unique group of neuropeptides, has emerged as a potentially important player in type 2 inflammation^12–15^. NmU was first identified from porcine spinal cord in 1985, with the suffix U referring to its stimulating effect on rat uterus^16^. Human NmU is a 25-amino acid peptide (hNmU-25) that shares the same C-terminal octapeptide with porcine NmU^17^. Two G-protein coupled receptors are thought to be the major receptors of NmU, designated as neuromedin U receptor 1 (NmUR1) and NmUR2^18^. NmUR1 is mainly detected in peripheral tissues such as small intestine and lung, while NmUR2 is highly expressed in specific regions of central nervous system (CNS)^18^. In addition to its roles in smooth muscle contraction, feeding and energy balance, NmU is also found to function as a pro-inflammatory driver in type 2 immune responses^19^. Murine Th2 cell line D10.G4.1 and mouse ILC2s express NmUR1, and NmU induces type 2 cytokine secretion from these cells in a NmUR1-dependent manner^12–15^. NmUR1 was also detected in murine eosinophil cell line Y-16, and treatment with NmU induced cell migration as well as adhesion to the components of the extracellular matrix^20^. Furthermore, NmU has been shown to promote airway eosinophilia in an allergic mouse model, as NmU-knockout attenuated the eosinophilic inflammation^20^. To date, however, all the functional studies of NmU in the immune system have been conducted in mice^21^. Although NmUR1 expression in human eosinophils, Th cells, and ILC2s has been detected^13,20^, the functional roles of NmU in human type 2 immunity have not yet been elucidated.

In this study, we investigated the expression of NmU in human blood and airways, the expression of NmUR1 in human immune cells, especially type 2 cells, and compared the expression in peripheral blood and lung tissue between healthy controls and patients with asthma. The effect of hNmU-25 alone and in the combination with other stimulators on these cells were also studied. Our observations provide evidence for a pathogenic role of neuropeptides in human type 2 immunity by demonstrating the involvement of hNmU-25 in promoting pro-inflammatory responses in human type 2 immune cells.

## Results

### NmU is expressed in human blood and airways

**Fig. 1:**
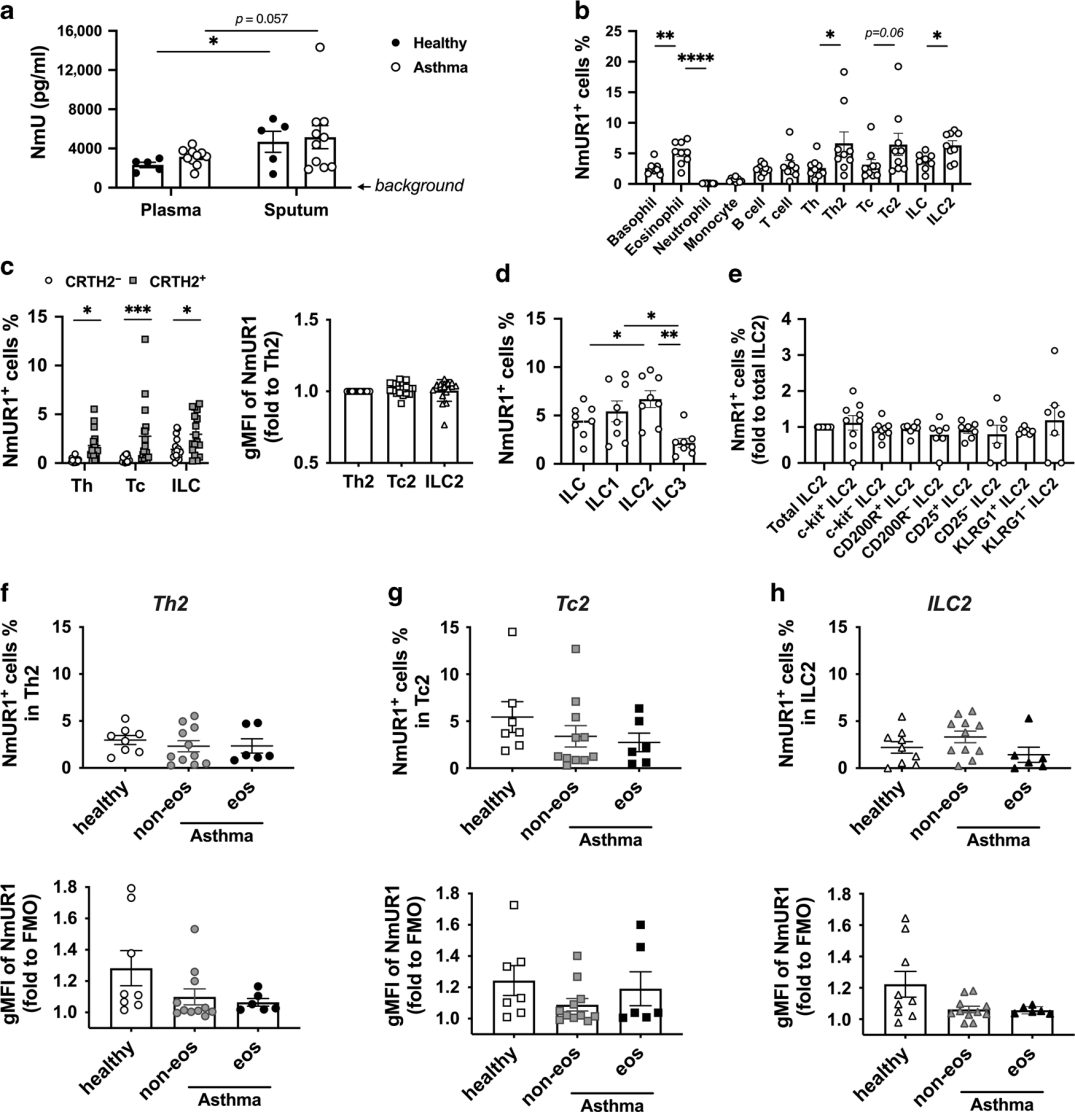
Expression of NmU and NmUR1 in human samples. **a** hNmU-25 levels in human plasma and sputum supernatants from healthy subjects or patients with asthma were detected by ELISA. **b** Comparison of NmUR1 expression in different human immune cells from fresh blood determined with flow cytometry. **c** NmUR1 expression in CRTH2^+^ (type 2 lymphocytes: Th2, Tc2 and ILC2) and CRTH2^-^ lymphocytes (other Th, Tc, and ILC). The expression levels in type 2 cells were compared with gMFI. **d**, **e** NmUR1 expression in ILC subsets (**d**) or in ILC2 subsets (**e**). **f**–**h** NmUR1 expression in Th2 (**f**), Tc2 (**g**) and ILC2 (**h**) cells in fresh blood among healthy controls (healthy), non-eosinophilic (non-eos) and eosinophilic (eos) asthma groups was compared. *p < 0.05, **p < 0.01, ***p < 0.001, ***p < 0.0001.

To investigate the role of NmU in asthma, we first addressed the question of whether NmU-25 is detectable in human samples. Blood and induced sputum were collected from healthy donors and patients with asthma. The concentration of hNmU-25 in plasma and sputum supernatants was determined with enyme-linked immunosorbent assay (ELISA) (Fig. 1a). hNmU-25 was detectable in both plasma and sputum supernatants, with slightly higher concentrations in the sputum. However, no significant difference in concentrations was detected between samples from healthy controls and patients with asthma.

### NmUR1 is expressed by human type 2 lymphocytes and upregulated in lung T cells

We next investigated the expression of NmU receptors in human immune cells from fresh blood (Fig. 1b). NmUR1 was detected in most immune cell types except neutrophils by flow cytometry. More NmUR1 positive cells were found in type 2 immune cells including Th2, Tc2, ILC2 cells and eosinophils. In contrast, NmUR2 was undetectable in these cells (Supplementary Fig. 1a).

In lymphocytes, NmUR1 expression was higher in the CRTH2 positive type 2 compartment compared to CRTH2 negative counterparts, although there was no significant difference among Th2, Tc2 and ILC2 cell types (Fig. 1c; Supplementary Fig. 2). In ILCs, NmUR1 expression was similar in ILC1s and ILC2s, while slightly lower in ILC3s (Fig. 1d). We also compared NmUR1 expression in different subsets of ILC2s^22,23^, but no significant difference was detected (Fig. 1e).

To investigate the expression of NmUR1 in asthma, we compared the levels of NmUR1 in type 2 lymphocytes between healthy individuals and patients with asthma (Supplementary Table 1). The expression levels of the receptor, in terms of the percentage of positive cells (Fig. 1f–h upper panels) and geometric mean fluorescence intensity (gMFI) (Fig. 1f–h lower panels), were similar between healthy controls, non-eosinophilic asthma and eosinophilic asthma groups.

**Fig. 2:**
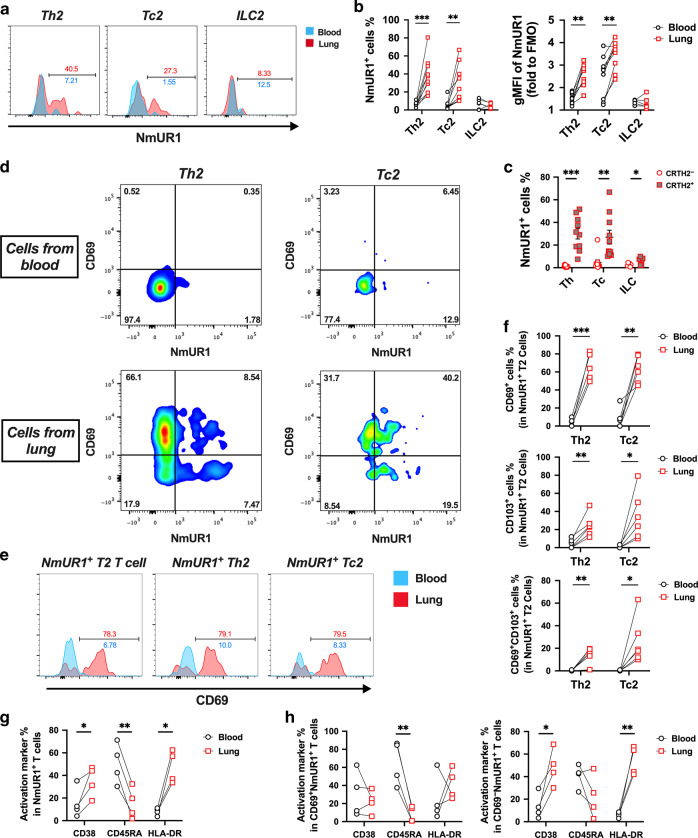
Expression of NmUR1 is upregulated in type 2 cells from lung. **a** Histograms and **b** individual value plots to compare NmUR1 expression in Th2, Tc2 and ILC2 cells from PBMCs and LMCs detected with flow cytometry. **c** Comparison of NmUR1 expression in CRTH2^+^ (Th2, Tc2 and ILC2) and CRTH2^-^ (other Th, Tc, and ILC) lymphocytes from lung tissues. **d** Increase of NmUR1^+^ cells in type 2 T cells from lung samples was mainly contributed by CD69^+^ cells. **e** Histograms to show the ratios of CD69 positive cells in different NmUR1^+^ type 2 cell groups. **f** Comparison of frequencies of CD69^+^, CD103^+^ or CD69^+^CD103^+^ Th2 or Tc2 cells in total NmUR1^+^ type 2 T cells between blood and lung. **g** CD38^+^ and HLA-DR^+^ cells were increased and CD45RA^+^ cells were decreased in the NmUR1^+^ T cells from LMCs. **h** The upregulation of CD38 and HLA-DR in the lung was mainly in CD69^-^ but not CD69^+^ cells. **a**, **d** and **e** are representative of 7 (**a**) or 6 (**d** and **e**) independent experiments. *p < 0.05, **p < 0.01 and ***p < 0.001.

We further compared the expression levels of NmUR1 in type 2 cells between the peripheral blood and the airways by using paired blood and resected lung tissues (Fig. 2). Flow cytometric analysis revealed that the levels of NmUR1 in lung-derived Th2 and Tc2 cells were higher than those in blood-derived samples (Fig. 2a, b). Expression levels in lung-derived ILC2s were comparable to those in blood. Similar to blood, the expression of NmUR1 in CRTH2^+^ type 2 compartments was significantly higher than that in CRTH2^-^ counterparts (Fig. 2c), although the level of NmUR1 in lung-derived CRTH2^-^ Th cells was also increased compared with blood (Supplementary Fig. 3a). To further investigate the mechanism of NmUR1 upregulation in the lung, we compared T cells derived from blood and lung tissues by staining the cells with tissue residency markers CD69 and CD103. The ratio of CD69^+^ T cells and CD69^+^CD103^+^ T cells was significantly increased in CD4^+^ and CD8^+^ T cells from lung (Supplementary Fig. 3b). The increase of NmUR1 positive cells in lung tissues appeared to be mainly attributable to CD69^+^ T cells, as the most significant enrichment of NmUR1^+^ Th2 and Tc2 cells detected in lung mononuclear cells (LMCs) was in CD69^+^ cells (Fig. 2d, e) but not CD103^+^ or CD69^+^CD103^+^ cells, although the proportion of all three groups (CD69^+^, CD103^+^ or CD69^+^CD103^+^) of cells in total NmUR1^+^ type 2 T, Th2 or Tc2 cells was increased in lung samples (Fig. 2 f). To determine whether the upregulation of NmUR1 in the lung tissues was due to cell activation, we also stained the cells from the lung tissues with cell activation markers CD38, CD45RA and HLA-DR. The levels of CD69 in NmUR1^+^ T cells in the lung were not associated with those of activation markers (Supplementary Fig. 4a, b), although the levels of CD38 and HLA-DR are slightly higher in CD69^+^NmUR1^+^ cells compared with NmUR1^-^ counterparts (Supplementary Fig. 4c). This suggests the upregulation of NmUR1 in the lung-resident memory type 2 T cells was not purely due to cell activation. However, we also observed an increase of CD38^+^ and HLA-DR^+^ cells and decrease of CD45RA^+^ cells in NmUR1^+^ T cells from the lung compared to blood (Fig. 2g). Although the latter changes were not associated with CD69 expression (Fig. 2h), these data taken together indicate that cell activation may contribute in part to the upregulation of NmUR1 in the lung.

### hNmU-25 induces human type 2 lymphocyte migration

**Fig. 3:**
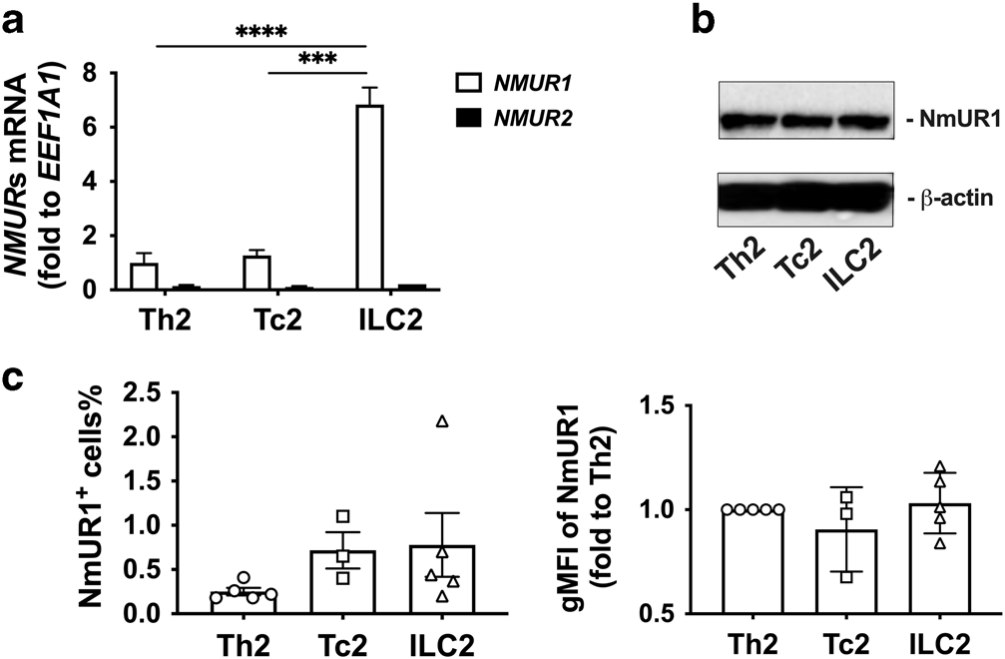
NmUR1 is expressed in cultured human type 2 lymphocytes. **a** mRNA levels of *NmUR1* and *NmUR2* in type 2 cells were compared with qRT-PCR. *EEF1A1* was used as a control gene. **b** Expression of NmUR1 in type 2 cells was detected with Western blot. β-actin was used as a control protein. **c** NmUR1 expression in type 2 lymphocytes was compared with flow cytometry. ***p < 0.001, ****P < 0.0001, (*n* = 4–8 for **a**; *n* = 3 for **b**).

To explore the role of NmU in human type 2 immunity, Th2, Tc2, and ILC2 cells were isolated from human blood and cultured for further in vitro investigation (Supplementary Fig. 2b). NmUR1 expression was detected in these cultured type 2 cells at both transcriptional (mRNA) and protein levels (Fig. 3a, b). In contrast, the expression of NmUR2 in the cultured type 2 cells was negligible (Fig. 3a and Supplementary Fig. 1b, c). No significant difference in expression levels of NmUR1 was observed among Th2, Tc2, and ILC2s (Fig. 3c), although higher mRNA level of *NmUR1* was detected in cultured ILC2s (Fig. 3a).

**Fig. 4:**
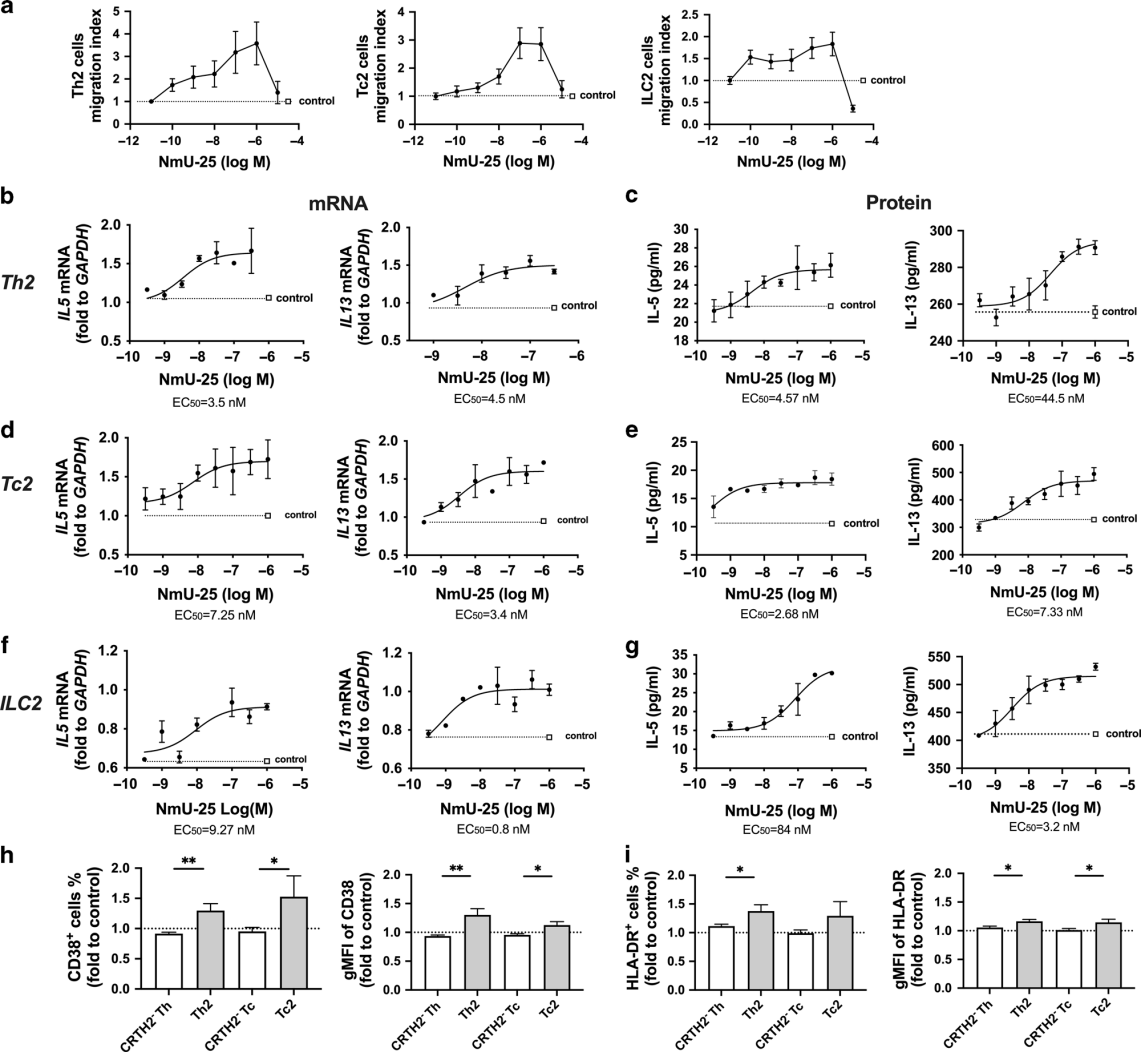
NmU induces activation of type 2 lymphocytes. **a** Th2, Tc2 and ILC2 cell migration to hNmU-25 measured with chemotaxis assays. **b**, **d**, **f** mRNA levels of *IL5* and *IL13* in cultured Th2 (**b**), Tc2 (**d**) and ILC2 (**f**) cells after treatment with various concentrations of hNmU-25 for 4 h detected with qRT-PCR. *GAPDH* was used as a control gene. **c**, **e**, **g** Protein levels of IL-5 and IL-13 in the supernatants of Th2 (**c**), Tc2 (**e**) and ILC2 (**g**) cultures after treatment with various concentrations of hNmU-25 for 4 h measured with ELISA. **h**, **i** Levels of CD38 (**h**) and HLA-DR (**i**) in CRTH2^-^ or CRTH2^+^ T cells in fresh PBMCs treated with 100 nM hNmU-25 determined with flow cytometry. *p < (*n* = 5–7 for **a**; *n* = 3–4 for **b**, **d** and **f**; *n* = 3–5 for **c**, **e** and **g**; *n* = 10 for **h**, **i**).

To understand whether the NmUR1 in these cultured type 2 lymphocytes was biologically functional, the Th2, Tc2, and ILC2 cells were treated with dose titrations of hNmU-25 in chemotaxis assays (Fig. 4a). hNmU-25 induced cell migration in a dose-dependent manner, exhibiting typical bell-shaped curves of chemotaxis peaking at approximately 1 µM in Th2 and ILC2s, and 100 nM-1 µM in Tc2 cells.

### hNmU-25 induces type 2 cytokine production in human type 2 lymphocytes

To further study the role of NmU in the activation of human type 2 lymphocytes, cultured cells were treated with serial concentrations of hNmU-25 for analysis of cytokine production. The transcriptional levels of *IL5* and *IL13* in the cells were measured by quantitative RT-PCR (qRT-PCR) (Fig. 4b, d, f), and the protein levels of IL-5 and IL-13 released in the cell supernatants were measured by ELISA (Fig. 4c, e, g). The treatment increased IL-5 and IL-13 levels at both transcriptional and protein levels in all three types of cells in a dose-dependent manner. The EC_50_ of hNmU-25 for *IL5* and *IL13* mRNA up-regulation in Th2 cells was 3.5 and 4.5 nM, in Tc2 cells was 7.25 and 3.4 nM, and in ILC2s was 9.27 and 0.8 nM, respectively (Fig. 4b, d, f). The EC_50_ of hNmU-25 for IL-5 and IL-13 production in Th2 cells was 4.57 and 44.5 nM, in Tc2 cells was 2.68 and 7.33 nM, and in ILC2s was 84 and 3.2 nM, respectively (Fig. 4c, e, g).

The stimulatory effect of hNmU-25 in human type 2 lymphocytes was confirmed ex-vivo in fresh PBMCs. hNmU-25 enhanced expression of activation markers CD38 and HLA-DR in Th2 and Tc2 cells but not in their CRTH2^-^ counterparts (Fig. 4h, i).

### hNmU-25 enhances type 2 cytokine production in human type 2 lymphocytes in response to PGD_2_ and alarmin stimulation

**Fig. 5:**
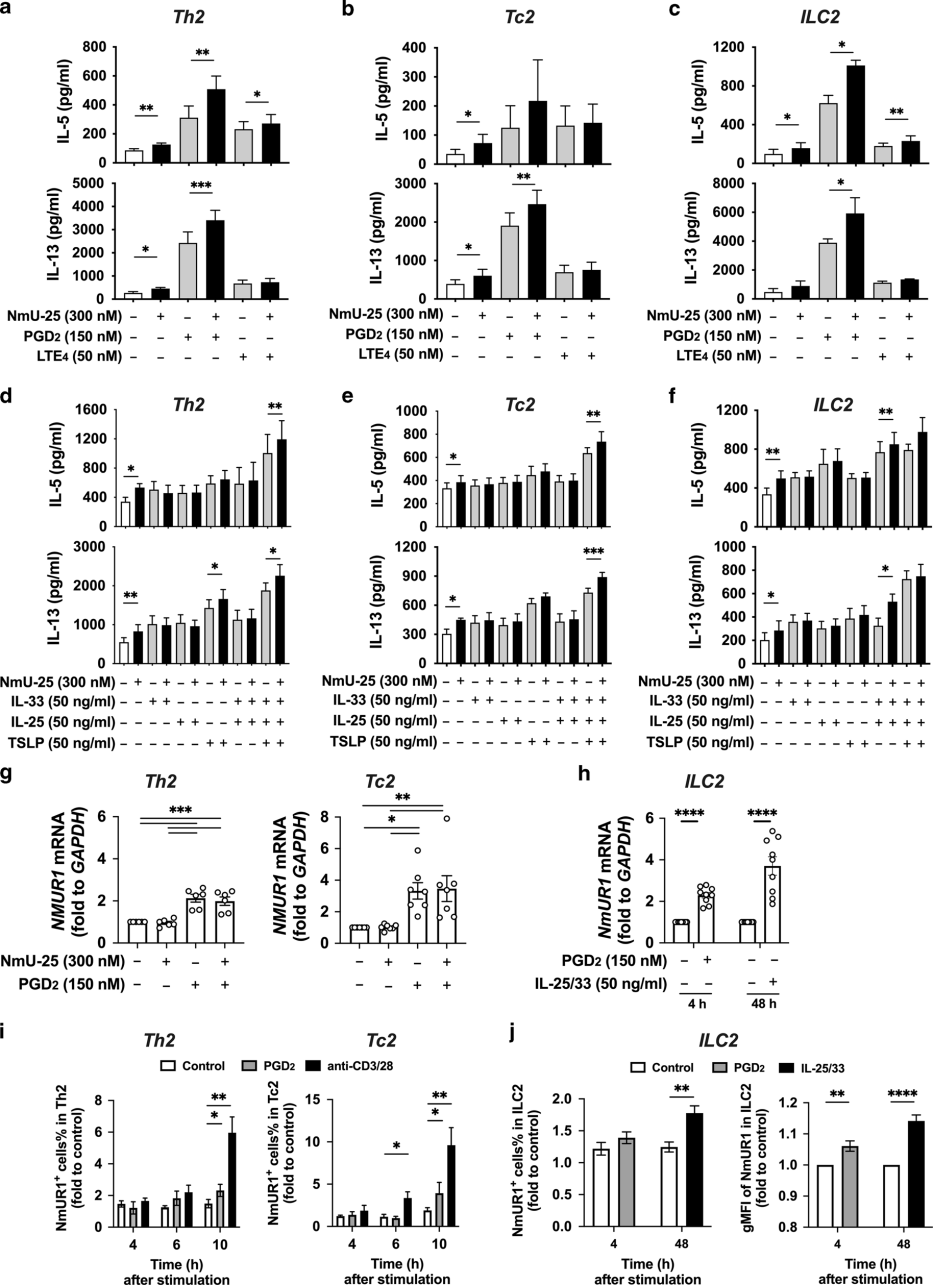
NmU enhances stimulatory effects of PGD2 and alarmin proteins in type 2 lymphocytes. **a-c** Levels of IL-5 and IL-13 in the supernatants of Th2 (**a**), Tc2 (**b**) and ILC2 (**c**) cultures after treatment with PGD_2_ or LTE_4_ alone or in combination with hNmU-25 for 4 h were measured with ELISA. **d**–**f** Levels of IL-5 and IL-13 in the supernatants of Th2 (**d**), Tc2 (**e**) and ILC2 (**f**) cultures after treatment with IL-33, IL-25 or TSLP alone or in combination with hNmU-25 for 48 h were determined with ELISA. **g, h** The levels of mRNA for *NMUR1* in cultured Th2 and Tc2 (**g**) or ILC2 (**h**) cells after stimulation with hNmU-25 and PGD_2_ or IL-25/33 measured with qRT-PCR. **i** NmUR1^+^ cells detected in cultured Th2 or Tc2 cells with flow cytometry at different time points after treatment with PGD_2_ or coated anti-CD3/28 antibodies. **j** NmUR1 expression in ILC2s after treatment with PGD_2_ (150 nM) or IL-25/33 (50 ng/ml). *p < 0.05, **p < 0.01, ***p < 0.001, ****p < 0.0001, (*n* = 7-10 for **a**, **b**, **d** and **e**; *n* = 4-5 for **c** and **f**; *n* = 9 for **i** and **j**).

As we previously reported, mast cell-derived lipid mediators, particularly PGD_2_ and LTE_4_, and alarmin proteins (IL-33, IL-25, and TSLP) are important stimulators of type 2 cells in asthma^7,24–26^. We next tested the effect of NmU on cytokine production in human Th2, Tc2, and ILC2 cells in response to these stimulators. Type 2 cytokine production in response to hNmU-25, PGD_2_, LTE_4_ and alarmins alone or their combination were compared in these cells (Fig. 5). The efficacy of hNmU-25 on type 2 cytokine production was weaker than that of PGD_2_, but similar to LTE_4_ alone (Fig. 5a–c). Synergistic enhancement of IL-5 and IL-13 production by the combination of PGD_2_ and hNmU-25 was observed in all three cell types except for IL-5 production by Tc2 cells. However, the enhancement of LTE_4_ stimulation was only observed in IL-5 production by Th2 and ILC2s. The efficacy of hNmU-25 on human type 2 lymphocytes was similar to that of alarmins alone. Moreover, no hNmU-25 effect was observed when hNmU-25 was combined with IL-25, IL-33 or TSLP individually to treat these cells (Fig. 5d–f). However, hNmU-25 additively enhanced IL-5 and IL-13 productions in response to the combination treatment with IL-25/IL-33/TSLP in Th2 and Tc2 cells or with IL-25/33 in ILC2s. These data indicated potential for NmU to enhance type 2 inflammation in asthma.

To understand the mechanism underlying the synergistic enhancement of hNmU-25 and PGD_2_, we examined the receptor expression after stimulation of the type 2 cells (Fig. 5g–j; Supplementary Fig. 5). hNmU-25 did not show any effect on the gene transcription of *PTGDR2* (encoding CRTH2) (Supplementary Fig. 5a). As expected, *PTGDR2* mRNA was slightly downregulated by PGD_2_ stimulation^24^. Upregulation of *NMUR1* transcription was detected after PGD_2_ stimulation (Fig. 5g, h). This upregulation was also observed at the protein level after stimulation with PGD_2_ for 10 h in Th2/Tc2 cells and for 4 h in ILC2s (Fig. 5i, j; Supplementary Fig. 5b). The upregulation of NmUR1 was even stronger in Th2/Tc2 cells via T cell receptor activation or in ILC2s via IL-25/33 stimulation (Fig. 5h–j).

### hNmU-25 induces eosinophil migration and degranulation

**Fig. 6:**
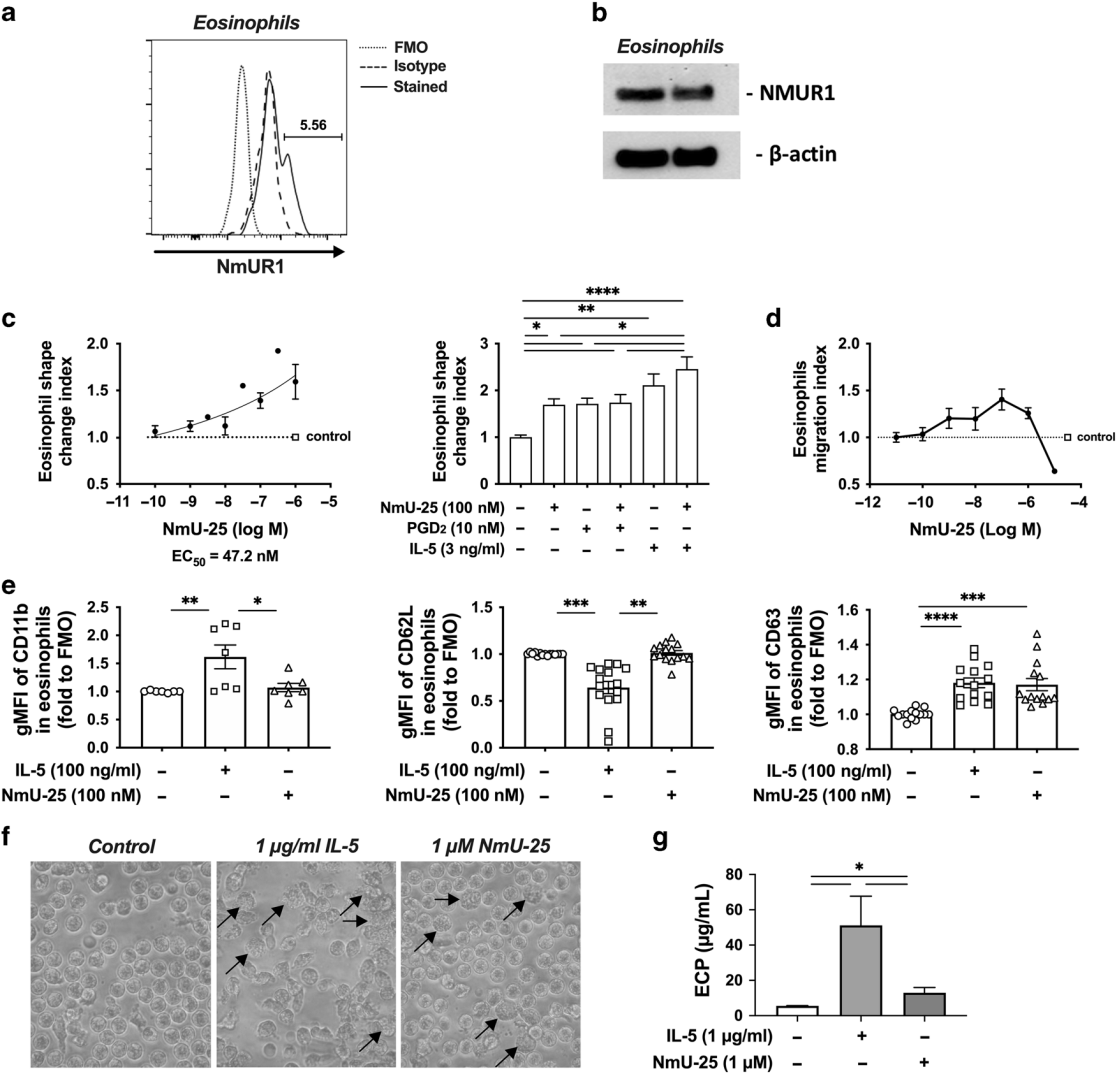
NmU induces eosinophil migration and degranulation. **a**, **b** NmUR1 expression in eosinophils was detected with flow cytometry (**a**) or Western blot (**b**). **c** Eosinophil shape-change induced by various concentrations of hNmU-25 or in combination with PGD_2_ or IL-5 measured with flow cytometry. **d** Eosinophil migration induced by various concentrations of hNmU-25 determined with chemotaxis assay. **e** Surface levels of CD11b, CD62L, and CD63 in eosinophils after treatment with hNmU-25 measured with flow cytometry. **f** Representative image of eosinophil degranulation induced by hNmU-25. **g** Levels of ECP in the supernatants of eosinophils after treatment with hNmU-25 for 4 h were measured with ELISA. IL-5 stimulation was used as a positive control. *p < 0.05, **p < 0.01, ***p < 0.001, ****P < 0.0001, (*n* = 3–5 for **c**; *n* = 4 for **d**; *n* = 5 for **g**).

Eosinophils play a critical pathogenic role in type 2 immunity-mediated diseases, such as eosinophilic asthma^27^. It has been reported that human eosinophils expressed NmUR1^20^. We confirmed NmUR1 expression in eosinophils by flow cytometry and Western blot (Fig. 6a, b). Treatment of fresh blood with hNmU-25 ex-vivo induced eosinophil shape change, a marker of eosinophil migration^28^, in a dose-dependent manner with EC_50_ = 47.2 nM (Fig. 6c left panel). hNmU-25 (100 nM) also enhanced eosinophil shape change induced by IL-5 (3 ng/ml) although no effect was detected on PGD_2_-induced eosinophil shape change (Fig. 6c right panel). To confirm the effect of hNmU-25 on eosinophil migration, chemotaxis assay using purified eosinophils was conducted (Fig. 6d). hNmU-25 induced eosinophil migration in a dose-dependent manner peaking at about 100 nM. To investigate the potential impact of NmU on other eosinophil functions, the levels of activation markers, CD11b and CD62L, and degranulation marker, CD63, in eosinophils were measured after treatment with hNmU-25 (Fig. 6e). CD11b and CD62L were unaltered, but CD63 was increased suggesting that NmU could be an inducer of eosinophil degranulation, as evidenced by the morphological changes of eosinophils (Fig. 6f) and the release of eosinophil cationic protein (ECP) after NmU treatment (Fig. 6g).

## Discussion

Given the worldwide prevalence of parasite infections and allergic diseases, identifying the underlying mediators in type 2 immune responses is beneficial for the development of novel therapies to manage these disorders. Several previous mouse or cell line-based studies have suggested that NmU could function as an important pro-inflammatory factor in type 2 inflammation, and this has expanded our appreciation of neuroimmune functions of NmU signalling^12,20^. To our knowledge, our study demonstrates for the first time the pro-inflammatory roles of NmU in human type 2 immunity. Increased levels of NmU and NmUR1 could be detected in human lung tissues and airways. NmU directly stimulated human type 2 lymphocytes to induce cell migration and type 2 cytokine production, and also induced eosinophil migration and degranulation. Importantly, NmU enhanced the responses of these type 2 cells to other pro-inflammatory triggers. These effects suggest a contribution to the pathogenic process of conditions associated with type 2 inflammation such as asthma.

NmU, a member of the neuromedin family, promotes inflammation in both neuron-dependent and neuron-independent manner^13,20,29^. NmU peptides are produced by different cell types such as sensory neurons, epidermal, endothelial cells and macrophages^13,29–31^, in multiple tissues and organs including the gastrointestinal tract and CNS^17,32^. In this study, we detected hNmU-25 in human blood and airways, with higher concentrations in the airways. It is still unclear which cell types are the major producers of NmU in human blood and airways. Human ILC2s have been demonstrated to express NmUR1^13^, indicating plausible role of NmU in this type of human cells. Our study not only confirmed this observation but also identified NmUR1 expression in other human lymphocytes, particularly CRTH2^+^ type 2 T cells that expressed higher levels of NmUR1 compared with non-type 2 cells. All the human type 2 lymphocyte subsets exhibited similar expression levels of NmUR1. Although we did not find significant differences between healthy controls and patients with asthma in terms of concentrations of hNmU-25 in the blood and airways, or levels of NmUR1 expression in type 2 cells, our paired blood and lung specimens from the same donors did reveal higher levels NmU in the airways and higher NmUR1 expression in the lung-derived type 2 T cells. These would suggest that the lung (airway) is likely an important location for NmU to exert its pro-inflammatory role in type 2 immunity in humans. The airways are the sites of pathogenic type 2 immune responses in allergic asthma, thus it will be interesting to investigate whether stimuli that result in eosinophilic airway inflammation such as allergen challenge promote NmU-25 release in human airways in future studies. Our data demonstrated that NmUR1 was upregulated not only in lung-resident Th2 and Tc2 cells, particularly CD69^+^ cells, but also in activated cells. Therefore, our results revealed a previously unknown regulation of NmUR1 expression by cell activation, which could happen during allergic immune response in asthma. Interestingly, the upregulation in the lung was not observed in ILC2s. Due to technical limitations in obtaining sufficient lung-resident type 2 cells for in vitro functional assays, we were unable to compare the hNmU-25 induced responses between paired blood and lung type 2 cells in this report. This will be addressed in future studies.

Among the major human NmU receptors, NmUR1 is expressed in different peripheral tissues^31,33^, whereas NmUR2 is preferentially expressed in specific regions of the CNS^18^. We only detected the expression of NmUR1 but not NmUR2 in human type 2 immune cells, which was consistent with a previous report on human ILC2s^13^. Although ILC2s showed significantly higher transcriptional level of *NmUR1* than Th2 and Tc2 cells by qRT-PCR, this was not matched by their protein levels. In in vitro functional studies, these three cell types behaved similarly in response to hNmU-25, which would suggest that the levels of functional receptor for hNmU-25 are similar.

The potential roles of NmU in type 2 inflammation have been reported in animal models. NmU elicited cytokine production in a murine Th2 cell line and enhanced mouse ILC2-mediated allergic pulmonary inflammation^12,15^. In this study, we demonstrated similar effects in human immune cells. Administration of hNmU-25 to cultured human type 2 cells not only induced cell migration, but also elicited type 2 cytokine production. More importantly, hNmU-25 enhanced the effect of other stimulators in these cells including lipid mediators (PGD_2_ and LTE_4_) and epithelial-derived alarmins (IL-25, IL-33, and TSLP)^7,26,34,35^. In a previous mouse study, the combination of NmU with IL-25 or IL-33 enhanced type 2 cytokine production from ILC2s^15^. Although we only detected marginal enhancement when hNmU-25 was combined with IL-25, IL-33 or TSLP alone, hNmU-25 showed significant enhancing effects when combined with the three alarmins simultaneously. Interestingly, hNmU-25 and PGD_2_ exhibited synergistic effects in type 2 cytokine production in all three types of type 2 lymphocytes. These data suggest that hNmU-25 could collaborate with alarmins and PGD_2_ to exert their pro-inflammatory roles in type 2 immunity. There is substantial evidence that the alarmins play key roles in driving type 2 inflammation in asthma. Damaged, stressed or infected epithelial cells produce alarmin cytokines^36^, and their levels in the airways are elevated in asthma^36–38^, which promotes type 2 cell activation. PGD_2_ is a major lipid mediator that is released from mast cells during an allergic response and is upregulated in asthma^39^. Through its high-affinity interaction with the receptor CRTH2, PGD_2_ elicits proinflammatory reactions in type 2 cells, including cell migration, cytokine production, and suppression of apoptosis^7,24,25,40^. Therefore, inhibition of alarmins or PGD_2_ are considered as potential approaches to treat type 2 immunity-mediated inflammatory diseases, which remain under clinical investigation^41–44^. Our data suggested that hNmU-25 could act collaboratively with alarmins and PGD_2_ to amplify type 2 immune responses in human diseases. It has been reported that pulmonary neuroendocrine cells amplify allergic asthma responses^11^. Therefore, under certain pathogenic conditions, hNmU-25/alarmins/PGD_2_-crosstalk could play an important role in type 2 inflammatory diseases.

The mechanism driving synergistic effects of hNmU-25 and PGD_2_ is still unclear. Upregulation of NmUR1 by PGD_2_ stimulation discovered in this study, could potentially contribute to the synergistic effect. However, this might not be the major driver of the synergy, as we were unable to detect the upregulation of NmUR1 at protein level at the time point (4 h) when the synergy was detected, except in ILC2s. Longer than 6 h seemed to be required to observe NmUR1 protein upregulation in the activated type 2 T cells. Both NmUR1 and CRTH2 are Gαi coupled receptors^45–47^. Their activation leads to Gαi-dependent intracellular Ca^2+^ mobilization and inhibition of cAMP accumulation. Ca2^+^-calcineurin-NFAT cascades, PI3K and MAPK signal pathways have been reported to be involved in NmUR1 or CRTH2-mediated cell signalling^12,48^. The common signaling pathways downstream of NmUR1 and CRTH2 could also be the underlying mechanism that drives the synergistic effect of hNmU-25 and PGD_2_. Further investigation into these signaling pathways would improve our understanding of the mechanism.

As an important part of type 2 immunity, eosinophils play critical roles in combating parasites and certain other infections in humans^49^, but they are also enriched and function as key effector cells in the pathogenesis of chronic inflammatory disorders of the airways including asthma^50^. It has been reported that human eosinophils express NmUR1, and NmU can induce eosinophil adhesion to extracellular matrix^20^. Here we confirmed the expression of NmUR1 in eosinophils, and also demonstrated a stimulatory effect of hNmU-25 on human eosinophils to induce cell-shape change, migration and degranulation. Although the effect of hNmU-25 was weaker than IL-5 and similar to PGD_2_, it enhanced eosinophil responses to IL-5. In this study, we did not detect an effect of hNmU-25 on the surface levels of CD11b and CD62L on eosinophils. However, it would be worthwhile to further investigate whether NmU possesses any effect on other markers of eosinophil activation. Current data indicate that NmU may contribute to the proinflammatory roles of eosinophils under certain pathogenic conditions.

In conclusion, NmUR1 is expressed by human immune cells with higher levels in type 2 lymphocytes including Th2, Tc2, and ILC2, and the expression levels are upregulated in lung-Th2 and Tc2 cells. The receptors are functional, as hNmU-25 not only elicits cell migration and type 2 cytokine production, but also enhances response to other stimulators, particularly PGD_2_. NmUR1 is also expressed by human eosinophils and mediates their migration and degranulation. Therefore, hNmU-25 could collaborate with alarmins and PGD_2_ to orchestrate type 2 immunity during disease development. Our findings revealed a neuroimmune regulatory pathway driven by the NmU/NmUR1 axis that potentially contributes to the pathogenesis of human type 2 airway inflammation, highlighting the axis as a candidate for new therapeutic intervention.

## Methods

### Human clinical samples

Patients meeting the American Thoracic Society/European Respiratory Society definition of asthma with a sputum eosinophil count of >3% (eosinophilic) or <3% (non-eosinophilic), and healthy control subjects were recruited from the John Radcliffe hospital, Oxford (Supplementary Table 1)^51^. Peripheral blood was collected and used directly for flow cytometry, cell culture preparation or plasma preparation. Donor-paired sputum was induced with nebulised saline solution (3–5%) after pre-treatment with salbutamol. Selected sputum plugs were dispersed with PBS, and supernatants were collected for ELISA analysis.

Samples for paired peripheral blood and resected lung tissues from the same individuals were collected by Oxford Radcliffe Biobank at John Radcliffe Hospital, Oxford on the day of surgery for lung cancer. The samples were used for paired blood-circulating and lung-resident type 2 cell comparison.

Ethical approval was granted by South Central-Oxford B Research Ethics Committee (18/SC/0361) and South Central-Oxford C Research Ethics Committee (19/SC/0173). Written informed consent was obtained from each donor before sample collection.

### Cell preparation and culture

Human Th2 and Tc2 cells were prepared from CD Leucocyte Cones (National Blood Service, Oxford, UK). In general, peripheral blood mononuclear cells (PBMCs) were prepared with a Lymphoprep gradient. CD3^+^CD4^+^CD8^-^CRTH2^+^ (Th2) or CD3^+^CD4^-^CD8^+^CRTH2^+^ (Tc2) cells were sorted from the PBMCs using antibody panel 1 (Supplementary Table 2) with the FACSAriaTM III sorter (BD Biosciences). ILC2s were prepared from healthy blood using the method modified from that described previously^24^. Briefly, CD3^+^ cells were pre-depleted from PBMCs by using CD3 microbeads (Miltenyi Biotec), and then the cells were labelled with antibody panel 2 (Supplementary Table 2). Lin^-^(CD3/4/8/11b/11c/14/16/19/56/123/FcεRI) CD45^high^CD127^+^CRTH2^+^ populations were sorted. All the cells (Th2, Tc2 and ILC2) were expanded in culture with RPMI containing 10% human serum, 1× L-glutamine, 1× penicillin/streptomycin, 1× sodium pyruvate, 1× MEM NEAA, 10 mM HEPES Buffer, 50 µM 2-mercaptoehanol and 250 U/mL IL-2 in the presence of irradiated feeder PBMCs.

Eosinophils were purified as previously described^7^. Briefly, an erythrocyte/granulocyte pellet was collected from fresh blood using Lymphoprep gradient. After 3% dextran (Sigma Aldirch) sedimentation for 30 min, the granulocyte supernatant was harvested. Remaining erythrocytes in the supernatant were lysed with 0.6 M KCl in water. Eosinophils were then purified from the granulocytes by negative selection with anti-human CD16 microbeads (Miltenyi Biotec). The cells with purity >80 % were used for further experiments.

### Flow cytometry

To detect NmUR1 expression in different immune cells (excluding ILCs) in vivo, fresh whole blood was labelled with antibody panel 3 (Supplementary Table 2), followed by red blood cell lysis with BD FACS lysing Solution (BD Biosciences), and in type 2 lymphocytes, ILC subsets or ILC2 subsets, PBMCs were labelled with antibody panel 4 or 5 respectively (Supplementary Table 2), including live/dead marker Zombie Aqua (BioLegend). Rabbit IgG antibody (Bioss Antibodies) was used as an isotype control for NmUR1 staining. To measure Th2/Tc2 cell activation ex-vivo, PBMCs were treated with or without 100 nM hNmU-25 (Phoenix Pharmaceuticals) for 24 h and then stained with antibody panel 6 (Supplementary Table 2).

For cultured type 2 lymphocytes, the cells were stained with antibody against NmUR1 and Zombie Aqua before or after stimulation with hNmU-25, PGD_2_ (Enzo Life Science), anti-CD3/28 antibodies (coated on plate, eBioscience) or IL-25/33 (BioLegend).

To measure eosinophil activation and degranulation, fresh blood was treated with an equal volume of RPMI medium containing hNmU-25 or IL-5 for 1 h at 37 °C. The treatment was stopped by washing the cells with PBS containing 2 mM EDTA. The samples were further labelled with antibody panel 7 (Supplementary Table 2) followed by red blood cell lysis with an RBC Lysis Solution.

The samples were acquired on a BD LSR II flow cytometer (BD Biosciences). Data were analyzed with FlowJo software (TreeStar). For some data, gMFI was calculated for each cell type individually including both stained and fluorescence minus one (FMO) samples.

### Western blotting

Protein was extracted from cultured type 2 cells or freshly isolated eosinophils using RIPA buffer (Sigma) containing 1% phosphatase inhibitor cocktail XI (Cambridge Bioscience, UK) and 1% protease inhibitor cocktail (Sigma). The concentrations of protein were determined with a BCA protein assay kit (Thermo Fisher Scientific). After normalization and denaturation, the samples were fractionated by SDS-PAGE and then electrophoretically transferred to a polyvinylidene difluoride membrane, followed by probing with antibodies against NmUR1 (Biorbyt, UK) and β-actin (CST). The results were recorded with ChemiDoc MP (Bio-Rad).

### qRT-PCR

Total RNA was extracted from cultured cells using a RNeasy Mini Kit (Qiagen). cDNA was prepared with a Reverse Transcription Kit (Invitrogen). qRT-PCR was conducted with the Master Mix and Probe (Roche) or Fast SYBR Green Master Mix (Applied Biosystems) in a CFX96^TM^ Real-Time System (Bio-Rad). *GAPDH* and *EEF1A1* were used as reference genes. Primers and probes used were listed in Supplementary Table 3.

### ELISA

The concentrations of hNmU-25 in paired human plasma or sputum from healthy donors or the patients with asthma were measured with the Human Neuromedin U ELISA Kit (MyBioSource) following the manufacturer’s instructions.

The concentrations of IL-5 and IL-13 in the supernatants of cultured type 2 cells or ECP in the supernatants of isolated eosinophils after treatment with hNmU-25 alone or in combination with PGD_2_, LTE_4_ (Enzo Life Science), or IL-25/33 and TSLP (BioLegend) were measured using ELISA kits (R&D Systems for IL-5/13 and Abcam for ECP). The results were detected by an EnVision Multilabel Plate Reader (PerkinElmer).

### Chemotaxis assay

Cultured type 2 cells or freshly purified eosinophils were resuspended in RPMI 1640 medium. 25 µl of cell suspension and 29 µl of hNmU-25 (various concentrations as indicated in the results) were loaded into the upper and lower chambers, respectively, in a 5 µm pore-sized ChemoTx plate (Neuro Probe). After incubation for 1 h at 37  °C, the migrated cells in the lower chambers were collected and measured with a Cell Titer-Glo Luminescent Cell Viability Assay (Promega), and quantified using an EnVision Multilabel Plate Reader.

### Eosinophil shape change assay

Fresh blood was incubated with an equal volume of RPMI 1640 medium containing various concentrations of hNmU-25 or in combination with IL-5, PGD_2_ for 1 h at 37 °C. The samples were fixed with a Cytofix Fixation Buffer (BD Bioscience) followed by red blood cell lysis using an RBC Lysis Solution (Gentra Systems). Eosinophil forward scatter was analysed on a BD LSRII flow cytometer.

### Paired blood-circulating and lung-resident type 2 cell comparison

Paired blood and lung tissues were processed within 24 h after collection. For blood samples, PBMCs were isolated using a Lymphoprep gradient. For lung tissues, specimens were cut into tiny pieces and then washed with DMEM containing 10% FCS to completely remove blood cells, followed by tissue digestion in DMEM containing 10% FCS, 10 µg/ml DNAse I, 1 mg/ml Collagenase D and 1 mg/ml Hyaluronidase (Sigma) at 37 °C for 45 min. Homogenized tissues were filtered through a 70 µm cell strainer and then incubated at 37 °C for a further 45 min. Single-cell suspension was obtained by passing through a 50 µm gauze, and then LMCs were isolated from the single-cell suspension using a Lymphoprep gradient.

PBMCs and LMCs were stained with antibody panel 8 for Th2 and Tc2 cells and panel 9 for ILC2s, or panel 10 for tissue-resident markers and panel 11 for cell activation markers (Supplementary Table 2) including Zombie Aqua. The samples were analysed and compared using a BD LSRII flow cytometer.

### Statistics

Data were analyzed with *t*-test (paired or unpaired) or one-way ANOVA followed by Tukey’s test. Data were presented as Mean±SEM. Values of *p* < 0.05 were regarded as statistically significant. All repeats indicated in the figures were independent biological repeats rather than technical replicates.

## Supplementary information

Supplementary Information
