# Supplementary material for: Neuromedin U promotes human type 2 immune responses
Source: Mucosal Immunol. 2022 Jul 9;15(5):990–9. doi: 10.1038/s41385-022-00543-6 (PMC9385483; doi:10.1038/s41385-022-00543-6)
Supplement: Supplementary file 1 — Supplementary Information [file 41385_2022_543_MOESM1_ESM.pdf]

**Neuromedin U promotes human type 2 immune responses**

Y Ye, J Luo, N Zeng, S Jiang, W Chen, RD Hoyle, P Klenerman, ID Pavord and L Xue

# Supplementary Information

## SUPPLEMENTARY METHODS

### Flow cytometry for NmUR2

To detect NmUR2 expression in type 2 lymphocytes, PBMCs were labelled with antibody panel 12 (Supplementary Table 2) or cultured Th2 and Tc2 cells were stained with NmUR2 antibody (Bioss Inc) including live/dead marker Zombie Aqua. The samples were acquired on a BD LSRII flow cytometer.

### Western blotting for NmUR2

Western blotting was conducted as described in the METHODS of the formal text. Antibody against NmUR2 was purchased from Novus.

## SUPPLEMENTARY TABLES

### Supplementary Table 1. Study subjects (Mean ± SD)

| Characteristic | Control (n=8) | Asthma Non-eosinophilic (n=11) | Asthma Eosinophilic (n=6) | *p*-value |
|---|---|---|---|---|
| Age (y) | 24.8 ± 3.5 | 53.8 ± 12.7 | 55.5 ± 12.4 | 0.0005 |
| Sex (male%) | 60 | 60 | 50 | |
| Atopy (%) | 0 | 50 | 50 | |
| BMI | 22.8 ± 2.9 | 29.1 ± 5.1 | 25.6 ± 1.3 | 0.04 |
| FEV1 (% pred) | 106.0 ± 15.0 | 84.1 ± 25.9 | 75.9 ± 4.2 | 0.09 |
| Sputum eosinophils (%) | 0.12 ± 0.25 | 0.2 ± 0.4 | 40.1 ± 47.5 | 0.07 |
| Blood eosinophils ($10^9$/L) | 0.084 ± 0.024 | 0.14 ± 0.114 | 0.593 ± 0.058 | <0.0001 |
| FeNO (ppb) | 19.0 ± 6.3 | 37.0 ± 15.1 | 62.8 ± 14.4 | <0.001 |

BMI, body mass index; FeNO, fractional exhaled nitric oxide; FEV1, forced expiratory volume in one second; SD, standard deviation.

**Supplementary Table 2. Antibodies used for flow cytometry and cell sorting***

| Antigen | Clone | Supplier | Antibody panels |
|---|---|---|---|
| CD3 | OKT3 | BioLegend | 1 |
| CD3 | UCHT1 | eBioscience | 2,4,12 |
| CD3 | SK7 | BioLegend | 2,3,5,6,8,9,10,11 |
| CD4 | RPA-T4 | BioLegend | 1 |
| CD4 | OKT4 | BioLegend | 2,3,4,5,6,8,9,10,11,12 |
| CD8 | SK1 | BioLegend | 1,3,4,6,8,10,11,12 |
| CD8 | RPA-T8 | BioLegend | 2,5,9 |
| CD11b | DCIS1/18 | Abcam | 2,4,5,9,12 |
| CD11b | ICRF44 | eBioscience | 7 |
| CD11c | BU15 | Life Technologies | 2,4,5,9,12 |
| CD14 | MφP9 | BD | 2,4,5,9,12 |
| CD14 | TuK4 | Life Technologies | 3 |
| CD16 | 3G8 | BioLegend | 2,4,5,7,9,12 |
| CD19 | HIB19 | BioLegend | 2,4,5,9,12 |
| CD19 | SJ25C1 | eBioscience | 3 |
| CD25 | BC96 | Biolegend | 5 |
| CD38 | HIT2 | BioLegend | 6,11 |
| CD45 | H130 | BioLegend | 2,4,5,9,12 |
| CD45RA | HI100 | BioLegend | 11 |
| CD56 | HCD56 | BioLegend | 2,4,5,9,12 |
| CD62L | DREG-56 | BioLegend | 7 |
| CD63 | H5C6 | BioLegend | 7 |
| CD69 | FN50 | BioLegend | 10,11 |
| CD103 | Ber-ACT8 | BioLegend | 10 |
| CD117 | 104D2 | BioLegend | 4 |
| CD123 | 32703 | R&D | 2,4,5,9,12 |
| CD127 | A019D5 | BioLegend | 2,4,5,9,12 |
| CD193 | 5E8 | BioLegend | 3 |
| CD200R | OX-108 | BD | 5 |
| CRTH2 | BM16 | Miltenyi Biotec | 1,2,3,4,5,6,8,9,10,11,12 |
| FcεRI | AER-37 (CRA-1) | BioLegend | 2,4,5,9,12 |
| HLA-DR | Tu36 | BioLegend | 6,11 |
| KLRG1 | SA231A2 | BioLegend | 5 |
| NmUR1 | Polyclonal | Bioss Inc | 3,4,8,9,10,11,12 |
| NmUR2 | Polyclonal | Bioss Inc | 12 |

*Panel 1 for T cell sorting; Panel 2 for ILC2 cell sorting; Panel 3-12 for flow cytometry.

**Supplementary Table 3. Primers and probes used for quantitative PCR**

| Gene | Primer | Probe no. |
|---|---|---|
| *EEF1A1* | F:5'- TCCTGTGAAACCCAGTGTCTT-3'<br>R:5'- TTCATTTATTGTAGTGAGCAAGTTTGT-3' | 62 |
| *GAPDH* | F:5'-AGCCACATCGCTCAGACAC-3'<br>R:5'-GCCCAATACGACCAAATCC-3' | SYBR Green |
| *IL13* | F:5'- AGCCCTCAGGGAGCTCAT-3'<br>R:5'- CTCCATACCATGCTGCCATT-3' | SYBR Green |
| *IL5* | F:5'- GGTTTGTTGCAGCCAAAGAT-3'<br>R:5'- TCTTGGCCCTCATTCTCACT-3' | SYBR Green |
| *NMUR1* | F:5'-CAGCCAGGTCCAGATACACC-3'<br>R:5'- ACACCACGACCAGGACAAA-3' | 67 |
| *NMUR2* | F:5'-TTTGTGGAGGAGTGGAGTGA-3'<br>R:5'-ATAATGGGGTTGACAGCTGAG-3' | 66 |
| *PTGDR2* | F:5'- CCTGTGCTCCCTCTGTGC-3'<br>R:5'-TCTGGAGACGGCTCATCTG-3' | SYBR Green |

## SUPPLEMENTARY FIGURES

**Supplementary Fig. 1**

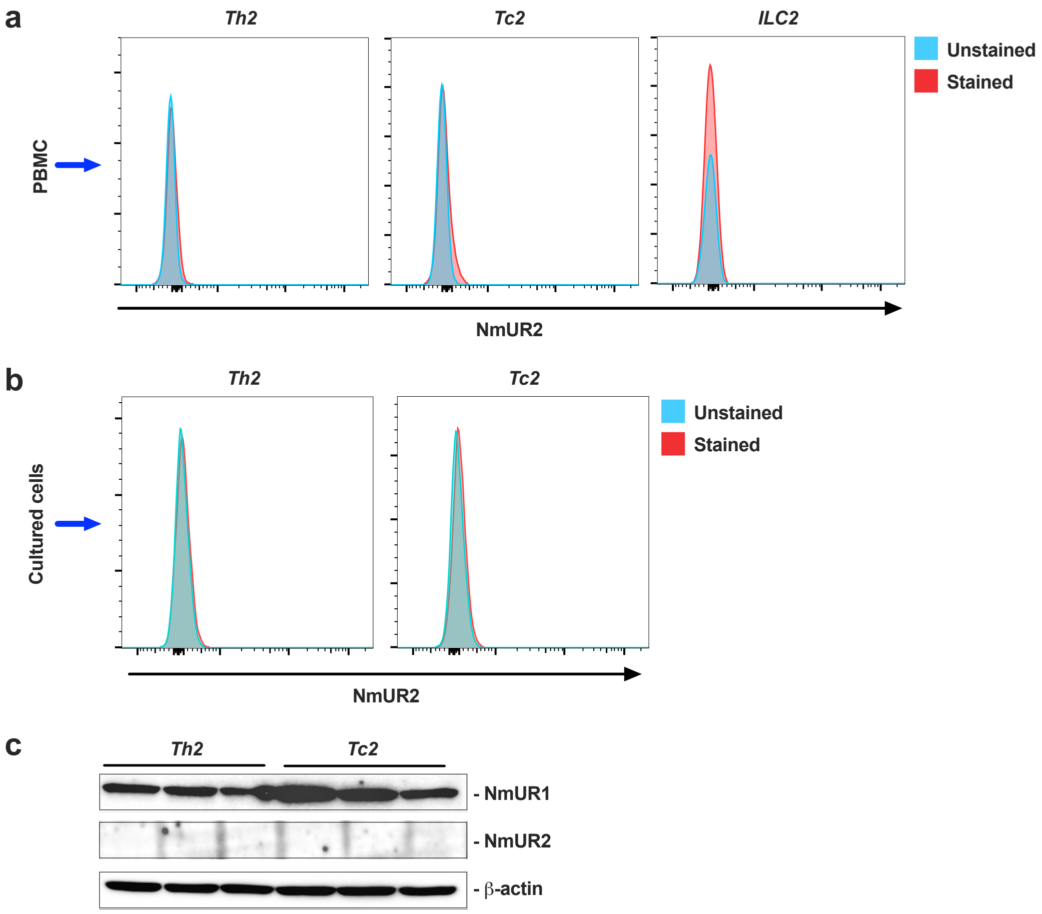


**Supplementary Fig. 1** Expression of NmUR2 was not detected in human type-2 lymphocytes. **a, b** Representative histograms of NmUR2 staining in Th2, Tc2 and ILC2 cells from fresh blood (**a**) or in cultured Th2 and Tc2 cells (**b**) measured with flow cytometry. **c** Expression of NmUR1 and NmUR2 in cultured Th2 and Tc2 cells were detected with Western blot. β-actin was used as a control protein.

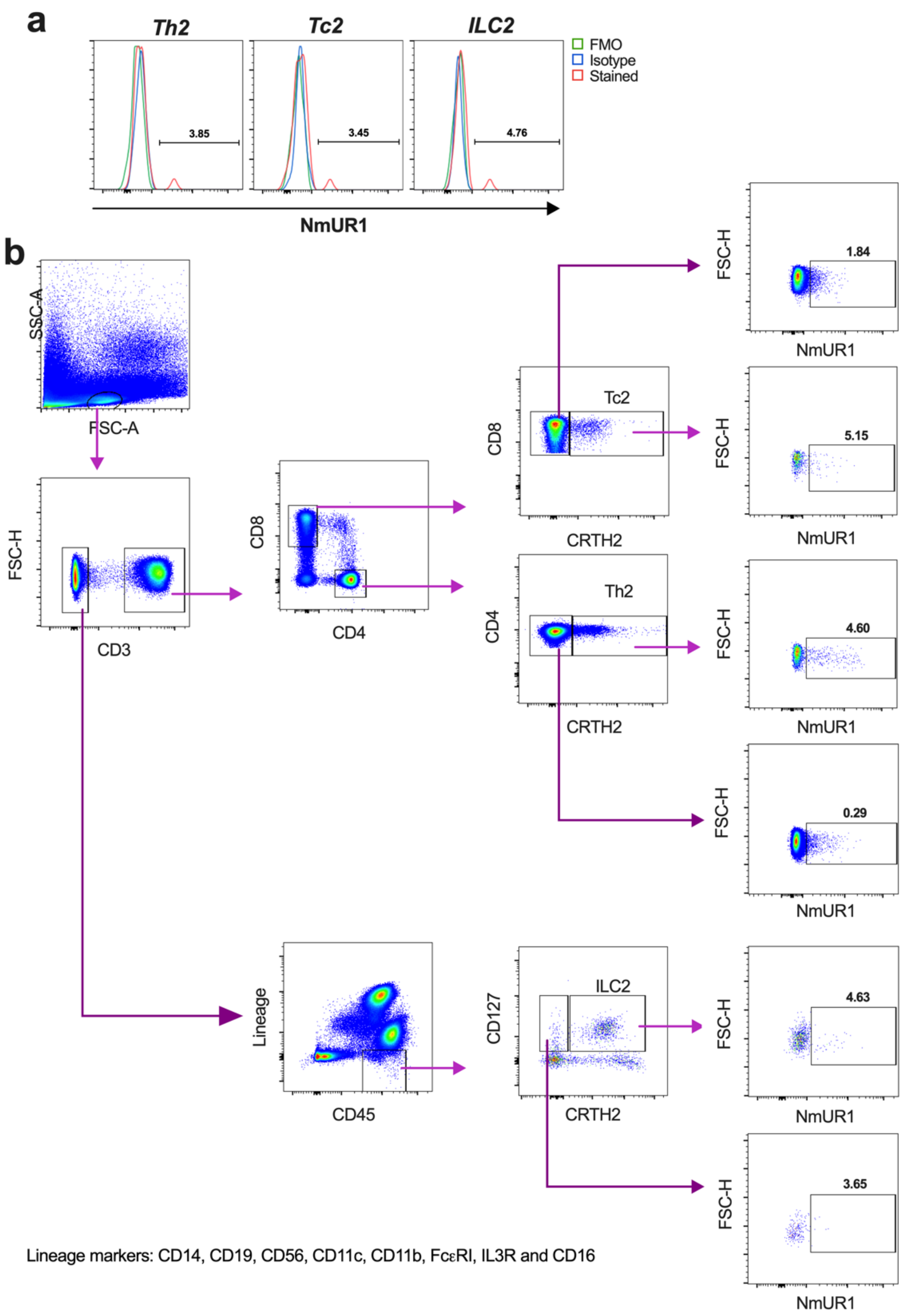


**Supplementary Fig. 2** Gating strategies for lymphocyte subsets and NmUR1 in peripheral blood from the Oxford cohort. Fresh blood was stained with a mixture of antibodies and analyzed with flow cytometry. **a** Representative histograms of NmUR1 staining in Th2, Tc2 and ILC2 cells. **b** Lymphocyte subsets were gated as Th2 ($CD3^+CD4^+CD8^-CRTH2^+$), CRTH2$^-$ Th ($CD3^+CD4^+CD8^-CRTH2^-$), Tc2 ($CD3^+CD4^-CD8^+CRTH2^+$), CRTH2- Tc ($CD3^+CD4^-CD8^+CRTH2^-$), ILC2 ($Lin^-CD3^-CD45^{high}CD127^+CRTH2^+$) and CRTH2$^-$ ILC ($Lin^-CD3^-CD45^{high}CD127^+CRTH2^-$) cells. Then NmUR1$^+$ cells were gated from these subsets of cells.

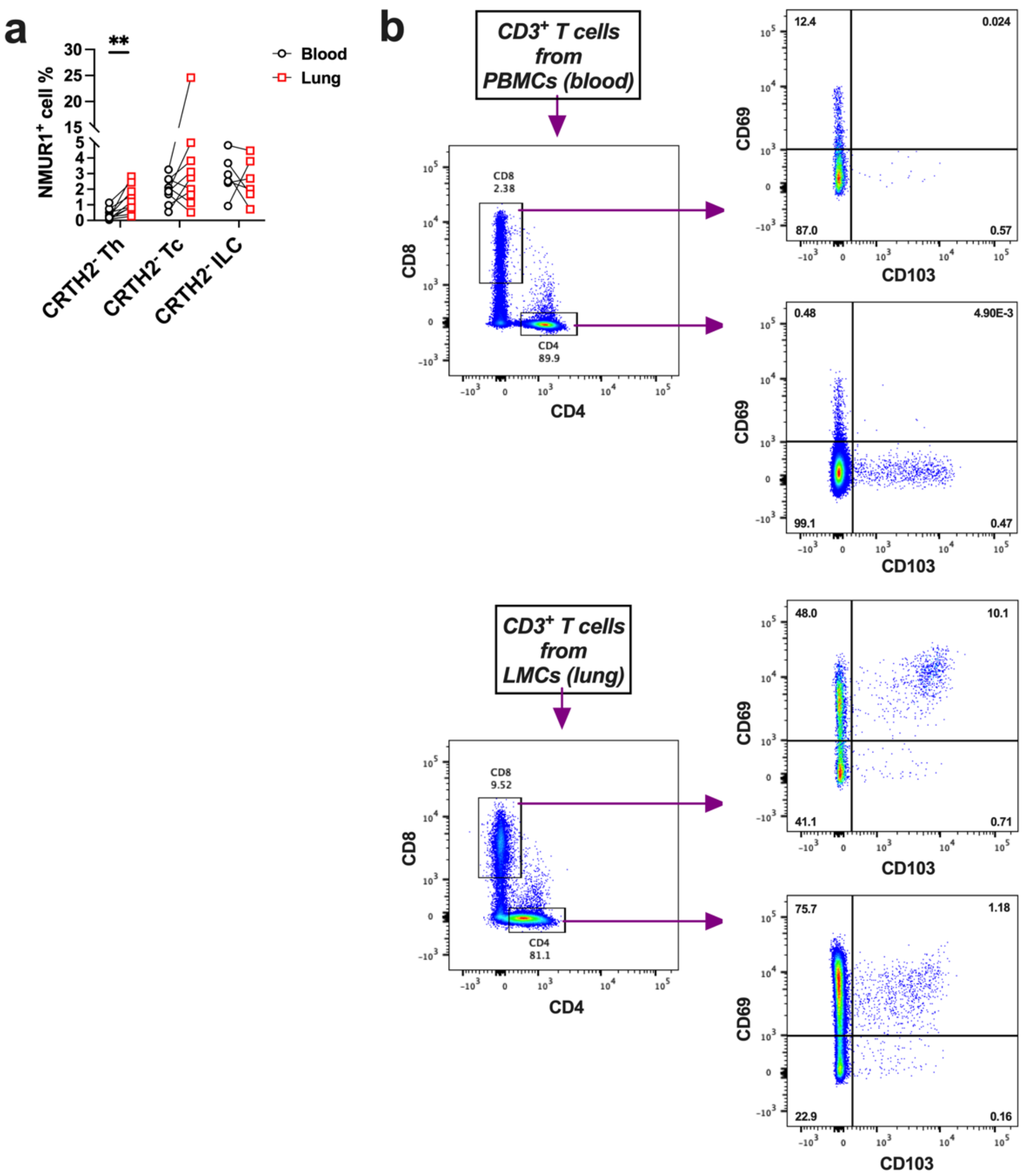


**Supplementary Fig. 3** Comparison of tissue resident T cells between paired blood and lung samples. **a** Comparison of NmUR1 expression in CRTH2$^-$ Th, Tc and ILC cells from blood PBMCs and lung LMCs detected with flow cytometry. **b** CD69$^+$ T cells and CD69$^+$CD103$^+$ T cells were enriched in both CD4$^+$ and CD8$^+$ T cells from LMCs. (**b**) showed a representative result of 6 paired samples. ** *p*<0.001.

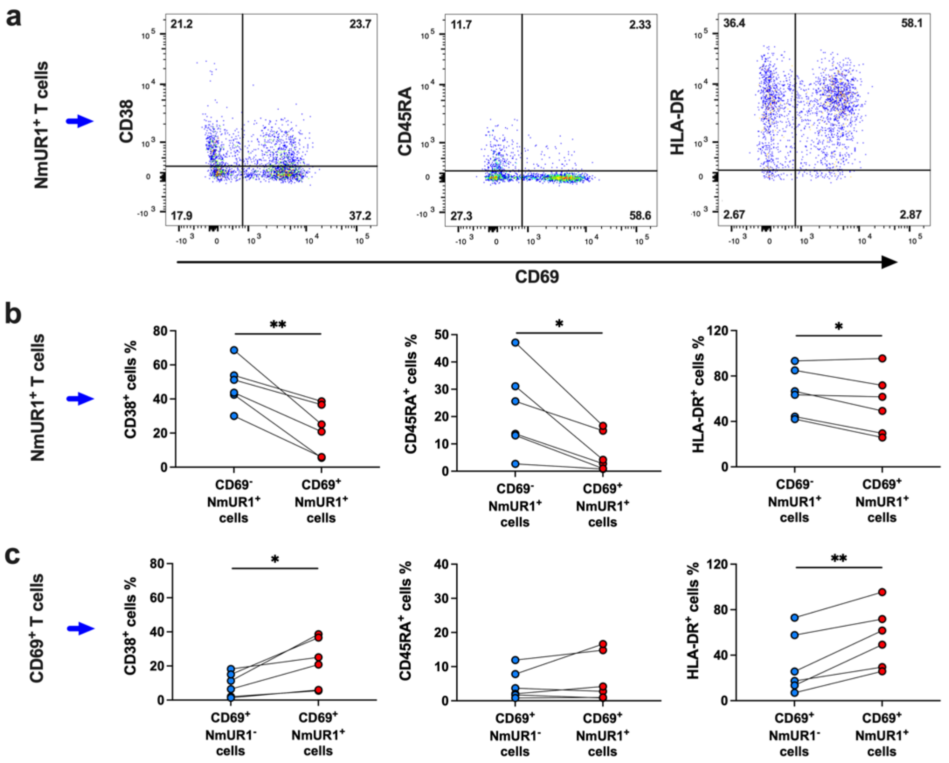


**Supplementary Fig. 4** Expression of activation markers in NmUR1$^+$ T cells from lung tissue. **a** Representative flow cytometric comparison of activation marker (CD38, CD45RA and HLA-DR) and CD69 staining in the cells from the lung samples. **b** Comparison of levels of activation markers between CD69$^-$ and CD69$^+$ NmUR1$^+$ T cells. **c** Comparison of levels of activation markers between NmUR1$^-$ and NmUR1$^+$ CD69$^+$ T cells. **a** is a representative of 5 independent experiments. * $p$<0.05, ** $p$<0.001.

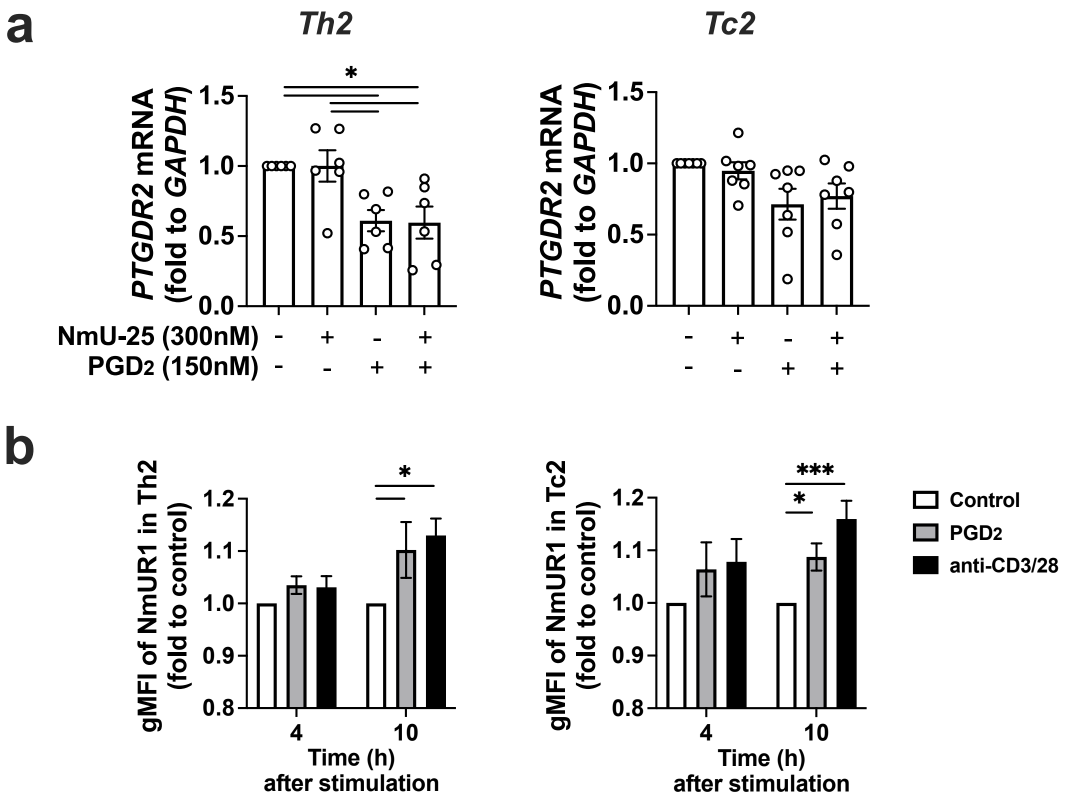


**Supplementary Fig. 5** Expression of CRTH2 and NmUR1 in cultured Th2 and Tc2 cells after stimulation. **a** The levels of mRNA for *PTGDR2* (encoding CRTH2) after stimulation with hNmU-25 and $PGD_2$ for 4 h measured with qPCR. **b** The levels of NmUR1 (gMFI) after treatment with $PGD_2$ (150 nM) or anti-CD3/28 antibodies (5 μg/ml) measured with flow cytometry. **p*<0.05, *** *p*<0.001, (n=9 for **b**).
